# ^225^Ac-iPSMA-RGD for Alpha-Therapy Dual Targeting of Stromal/Tumor Cell PSMA and Integrins

**DOI:** 10.3390/ijms242316553

**Published:** 2023-11-21

**Authors:** Blanca Ocampo-García, Pedro Cruz-Nova, Nallely Jiménez-Mancilla, Myrna Luna-Gutiérrez, Rigoberto Oros-Pantoja, Nancy Lara-Almazán, Diana Pérez-Velasco, Clara Santos-Cuevas, Guillermina Ferro-Flores

**Affiliations:** 1 Department of Radioactive Materials, Instituto Nacional de Investigaciones Nucleares, Ocoyoacac 52750, Mexico; pedro.cruz.comecyt@inin.gob.mx (P.C.-N.); myrna.luna@inin.gob.mx (M.L.-G.); nancy.l.servicios@inin.gob.mx (N.L.-A.); guillermina.ferro@inin.gob.mx (G.F.-F.); 2 Cátedras, CONACyT, Instituto Nacional de Investigaciones Nucleares, Ocoyoacac 52750, Mexico; nallely.jimenez@inin.gob.mx; 3 Faculty of Medicine, Universidad Autónoma del Estado de México, Toluca 50180, Mexico; rigoberto_ros@hotmail.com; 4 Faculty of Chemistry, Universidad Autónoma del Estado de México, Toluca 50180, Mexico; dperez540@alumno.uaemex.mx

**Keywords:** actinium-225, PSMA, RGD, dual-targeting, alpha-therapy, PSMA-RGD

## Abstract

Prostate-specific membrane antigens (PSMAs) are frequently overexpressed in both tumor stromal endothelial cells and malignant cells (stromal/tumor cells) of various cancers. The RGD (Arg-Gly-Asp) peptide sequence can specifically detect integrins involved in tumor angiogenesis. This study aimed to preclinically evaluate the cytotoxicity, biokinetics, dosimetry, and therapeutic efficacy of ^225^Ac-iPSMA-RGD to determine its potential as an improved radiopharmaceutical for alpha therapy compared with the ^225^Ac-iPSMA and ^225^Ac-RGD monomers. HEHA-HYNIC-iPSMA-RGD (iPSMA-RGD) was synthesized and characterized by FT-IR, UV-vis, and UPLC mass spectroscopy. The cytotoxicity of ^225^Ac-iPSMA-RGD was assessed in HCT116 colorectal cancer cells. Biodistribution, biokinetics, and therapeutic efficacy were evaluated in nude mice with induced HCT116 tumors. In vitro results showed increased DNA double-strand breaks through ROS generation, cell apoptosis, and death in HCT116 cells treated with ^225^Ac-iPSMA-RGD. The results also demonstrated in vivo cytotoxicity in cancer cells after treatment with ^225^Ac-iPSMA-RGD and biokinetic and dosimetric properties suitable for alpha therapy, delivering ablative radiation doses up to 237 Gy/3.7 kBq to HCT116 tumors in mice. Given the phenotype of HCT116 cancer cells, the results of this study warrant further dosimetric and clinical studies to determine the potential of ^225^Ac-iPSMA-RGD in the treatment of colorectal cancer.

## 1. Introduction

Human tumors exhibit intrinsic heterogeneity and changes in phenotype during the progression of the disease, implying different levels of expression of cell surface receptors. Dual targeting approaches can potentially improve therapeutic outcomes by targeting multiple receptors involved in disease progression. Heterodimeric molecules selectively address pathological cells to allow specific imaging or therapy. The α_v_β_3_ integrin is one of the most studied integrins, and represents a particular biomarker for targeting angiogenesis in the tumoral stroma. Likewise, α_v_β_3_ integrins can specifically be detected in vivo using labeled RGD (Arg-Gly-Asp) peptides [1]. PSMA (prostate-specific membrane antigen) expression has been found in the neovasculature of various cancers, including prostate, gynecologic, and soft tissue tumors [2]. The overexpression of this protein is not limited to the cell surface of prostate cancer epithelial cells since it is also found in nonprostatic tumors, including the epithelial cancer cells of melanoma and small cell lung cancer, hepatocellular cancer, cholangiocarcinoma, rectal carcinoma, glioblastoma, and breast cancer, among others [3,4,5]. In particular, colorectal cancer cells express PSMA and integrins subunit alpha v and subunit beta 3 [6].

Alpha particles have a higher linear energy transfer (LET) than beta particles, allowing for a larger fraction of energy to be deposited in cells, increasing therapeutic effectiveness. Additionally, the short range of alpha radiation in human tissue (50–100 μm) effectively kills targeted cancer cells while limiting damage to surrounding healthy tissue [7]. ^225^Ac (actinium-225) is considered a promising candidate for targeted alpha therapy due to its relatively long half-life (9.9 days) and yield of four alpha particles in chain decay (Fr-221, At-217, Bi-213, Po-213) with 27.6 MeV total energy (in the range of 5.8 to 8.4 MeV) with associated tissue ranging from 47 to 85 μm. Additionally, the decay chain produces two beta particles and two gamma emissions from the decay of Fr-221 (218 keV) and Bi-213 (440 keV) [8,9,10].

Several peptides, antibodies, and small molecules have been radiolabeled with ^225^Ac. Clinical results with ^225^Ac-PSMA-617 and ^225^Ac-PSMA-I&T demonstrated promising antitumor effects in patients with metastatic castrate-resistant prostate cancer [11,12]. ^225^Ac-RGD has also demonstrated in vivo efficacy therapy against α_v_β_3_-positive tumors [13,14]. However, ^225^Ac-therapy presents several limitations, where the main one is the occurrence of xerostomia and, to a minor extent, leucopenia, anemia, thrombocytopenia, and nephrotoxicity, which can be derived in nephropathy [12,15,16,17]. These side effects have been reduced by the co-administration of low activities of ^225^Ac-PSMA and ^177^Lu-PSMA [18,19]. A recent report showed that combining alpha emitters with betta emitters may mitigate significant adverse effects in comparison to alpha monotherapy, increasing, in consequence, the therapeutic efficacy. Methods to minimize organ toxicity are needed to treat patients safely and preserve their quality of life. Since cancer cells show upregulation of more than one receptor, a bispecific peptide addressing two target proteins in tumors may significantly improve targeted radiotherapy.

Since PSMA is frequently overexpressed in the tumor microenvironment and tumor cells (stromal/tumor cells) of various cancers, and RGD can specifically detect integrins involved in the tumoral angiogenesis, it is expected that a combination of PSMA inhibitor and RGD peptides in one molecule labeled with ^225^Ac improves the response to the alpha-targeted radiotherapy attributed to the synergistic recognition of PSMA and integrins.

This study aimed to preclinically evaluate the cytotoxicity, biokinetics, dosimetry, and therapeutic efficacy of ^225^Ac-iPSMA-RGD to determine its potential as an improved radiopharmaceutical for alpha therapy compared with the ^225^Ac-iPSMA and ^225^Ac-RGD monomers.

## 2. Results and Discussion

### 2.1. Synthesis and Chemical Characterization of HEHA-HYNIC-iPSMA-RGD (iPSMA-RGD)

**Figure A1:**
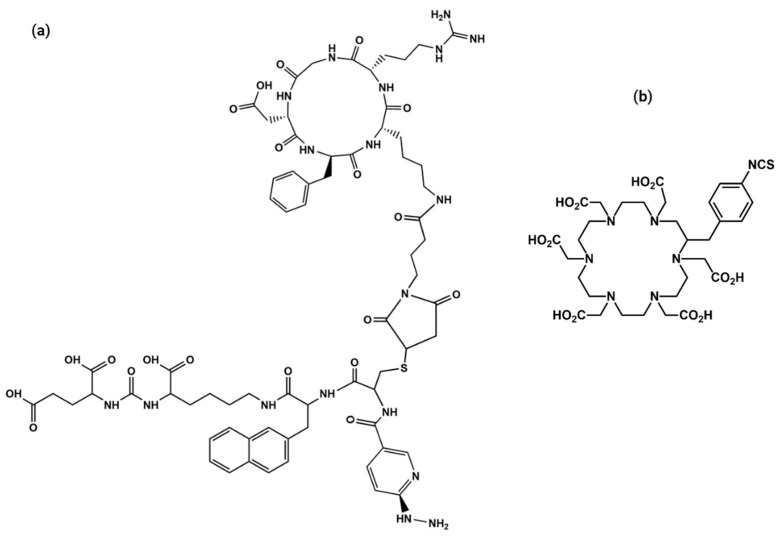
Schematic chemical structure of (**a**) HYNIC-iPSMA-RGD, (**b**) p-SCN-benzene-HEHA (HEHA).

**Figure A2:**
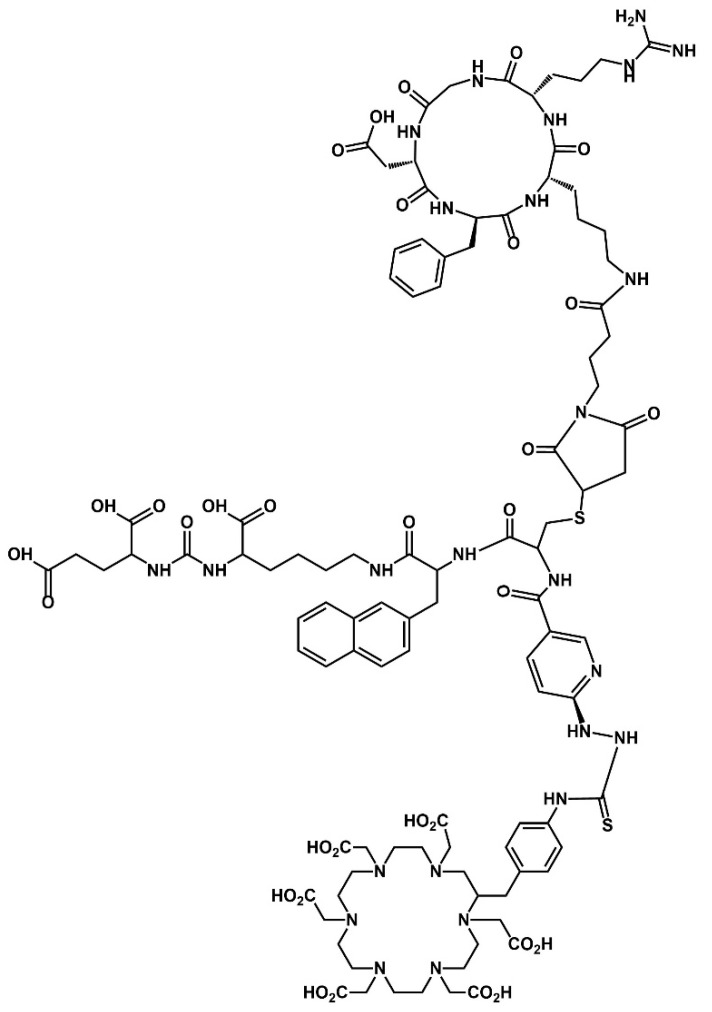
Schematic chemical structure of HEHA-HYNIC-iPSMA-RGD (iPSMA-RGD).

The schematic chemical structures of p-SCN-benzene-HEHA, HYNIC-iPSMA-RGD, and HEHA-HYNIC-iPSMA-RGD (iPSMA-RGD) are shown in Appendix A (Figure A1 and Figure A2). The iPSMA-RGD dimeric peptide was designed in this form to generate a potential ^99m^Tc-HYNIC-iPSMA-RGD/^225^Ac-HEHA-HYNIC-iPSMA-RGD (^225^Ac-iPSMA-RGD) theranostic pair. The chemical synthesis was based on the coupling reaction of p-SCN-benzene-HEHA with the HYNIC-iPSMA-RGD peptide. After HPLC purification, the chemical characterization was performed by FT-IR spectroscopy, mass spectrometry, and UV-Vis spectroscopy.

**Figure 1:**
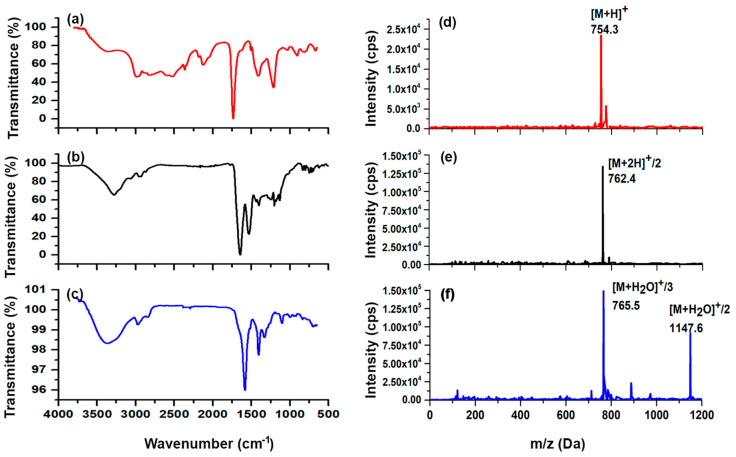
Infrared spectra of (**a**) p-SCN-benzene-HEHA (HEHA), (**b**) HYNIC-iPSMA-RGD, and (**c**) HEHA-HYNIC-iPSMA-RGD (iPSMA-RGD). Mass spectra of (**d**) HEHA, (**e**) HYNIC-iPSMA-RGD, and (**f**) HEHA-HYNIC-iPSMA-RGD (iPSMA-RGD).

The vibrational frequencies of the main functional groups observed in p-SCN-Benzene-HEHA (Figure 1a) and HYNIC-iPSMA-RGD (Figure 1b) were also present in HEHA-HYNIC-iPSMA-RGD (Figure 1c). In HYNIC-iPSMA-RGD, the C=O stretching of amide I was observed at 1646 cm^−1^ and N-H bending at 1555 cm^−1^. The absence of the NH_2_ band from the hydrazine group and the new thiourea bond was observed at 2127 cm^−1^ (p-SCN-Benzene-HEHA), which disappeared on HEHA-HYNIC-iPSMA-RGD, demonstrating the functionalization of the chelating agent to the HYNIC-iPSMA-RGD, as can be observed in Figure 1. In addition, FT-IR analysis of the conjugate showed that the band at 1646 cm^−1^ from the C=O stretching mode of carbonyl bonds (-CO-NH-) decreased in intensity and energy to 1642 cm^−1^ in HEHA-HYNIC-iPSMA-RGD. The characteristic vibrational bands at 1662, 1583, and 1404 cm^−1^ assigned to amide I, II, and III, respectively, from the heterodimeric HYNIC-iPSMA-RGD peptide were observed with slight shifts in the HEHA peptide conjugate. The O–H stretch bond of carboxylic acid was centered at 3351 cm^−1^ [20].

Mass spectrometry also corroborated the starting molecule and final product identity. As can be observed in Figure 1d, p-SCN-benzene-HEHA showed a *m*/*z* of 754.3 (calculated 753.3) from [M + H]^+^. HYNIC-iPSMA-RGD *m*/*z* of 762.4 [M + 2H]^+^/2 is also shown in Figure 1e. The hydrated form of HEHA-HYNIC-iPSMA-RGD peptide was observed as a consequence of the solute-solvent interaction with *m*/*z* of 1147.6 [M + H_2_O]^+^/2 and *m*/*z* of 765.5 [M + H_2_O]^+^/3, confirming the correct conjugation of the -NH_2_ group from hydrazine to the isothiocyanate group of p-SCN-benzene-HEHA (Figure 1f).

**Figure 2:**
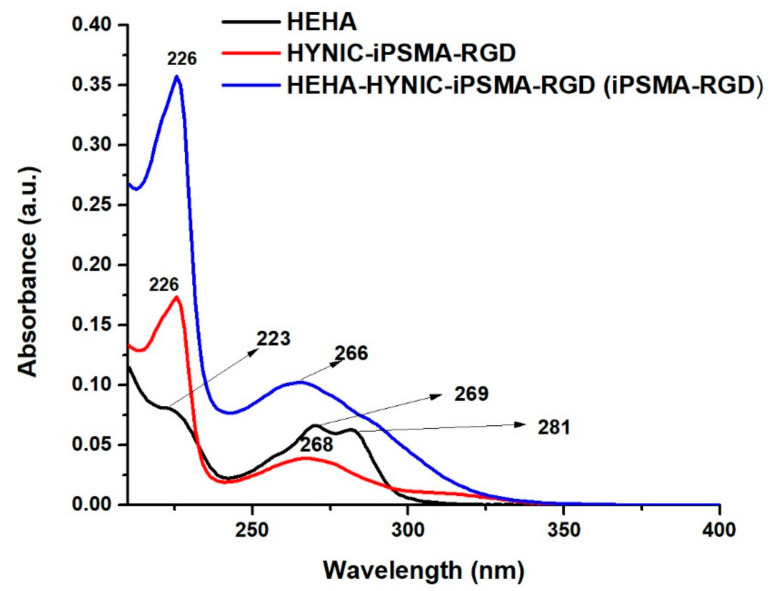
UV-Vis spectra of p-SCN-benzene-HEHA (HEHA), HYNIC-iPSMA-RGD, and HEHA-HYNIC-iPSMA-RGD (iPSMA-RGD).

In Figure 2, the UV-Vis spectra of p-SCN-benzene-HEHA, HYNIC-iPSMA-RGD, and iPSMA-RGD display absorption bands centered at 226 and 223 nm, indicating $n\to \pi^{*}$ transition of C=O bonds and the $\pi \to \pi^{*}$ transition of the aromatic C=C bond. The $n\to \pi^{*}$ transition was observed at 269 and 281 nm for p-SCN-benzene-HEHA and 268 nm for the heterodimeric peptide. Since the amine group of hydrazine from HYNIC-iPSMA-RGD was used for the coupling reaction, after conjugation, the heterodimeric peptide showed a broad absorption band with enhanced intensity centered at 266 nm and a shoulder at 288 nm, with bathochromic and hypochromic shift due to the presence of distinct chemical substituents on the organosulfur compound (isothiocyanate-NCS group).

### 2.2. Preparation of ^225^Ac-iPSMA-RGD

**Figure 3:**
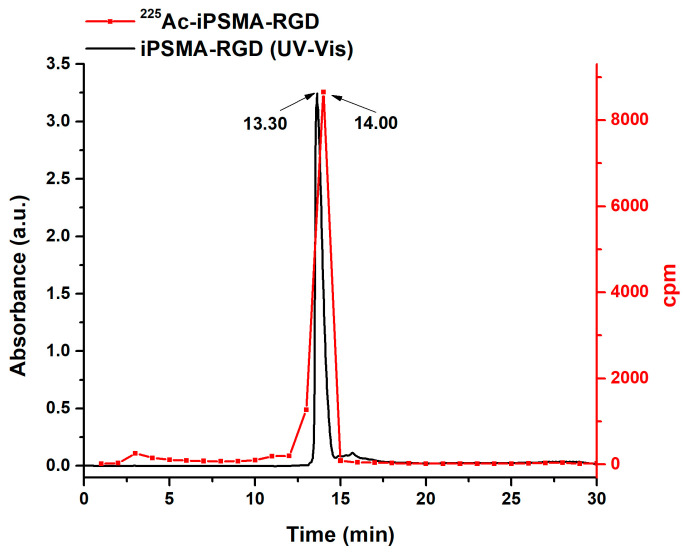
Reversed-phase UV-Vis-HPLC chromatogram (λ = 270 nm; 0.1 mg/mL of iPSMA-RGD) and radio-HPLC chromatogram of ^225^Ac-iPSMA-RGD graphed from the collected fractions measured in an external radioactive detector.

^225^Ac-iPSMA-RGD was initially obtained with radiochemical purities of 87.5 ± 3.5%, which increased to 96.3± 2.4% after purification by solid-phase extraction (Sep-Pak C18 cartridges) as determined by radio-HPLC (C-18 reversed phase) eluted fractions (1 mL) counted in a NaI(Tl) detector (Figure 3). The retention time for ^225^AcCl_3_ was 3.0–3.5 min and 14.0–14.5 min for ^225^Ac-iPSMA-RGD. The agreement of retention times between the iPSMA-RGD peak observed in the UV-Vis chromatogram and the ^225^Ac-iPSMA-RGD radio-chromatogram is proof of the radiopharmaceutical chemical identity (Figure 3) [21].

### 2.3. In Vitro Evaluation of ^225^Ac-iPSMA-RGD

#### 2.3.1. Stability in Saline Solution (0.9% NaCl) and Human Serum

The binding of proteins, especially human serum albumin, can contribute to the toxicity of the radiopharmaceutical by inhibiting its uptake and internalization at the target site in vivo. Since the diffusion rate of the radiopharmaceutical from blood to target tissues can be limited by protein binding [22], the stability of ^225^Ac-iPSMA-RGD in serum was evaluated. Radiopharmaceutical binding protein was 7.7 ± 0.8% after 3 h. However, the radiochemical purity of ^225^Ac-iPSMA-RGD decreased by 2%, 10%, and 30% at 5, 24, and 72 h, respectively, after preparation in both saline and serum. Therefore, for clinical applications, ^225^Ac-iPSMA-RGD must be used in less than five hours after preparation.

#### 2.3.2. Cellular Uptake, Internalization, and Viability

**Figure 4:**
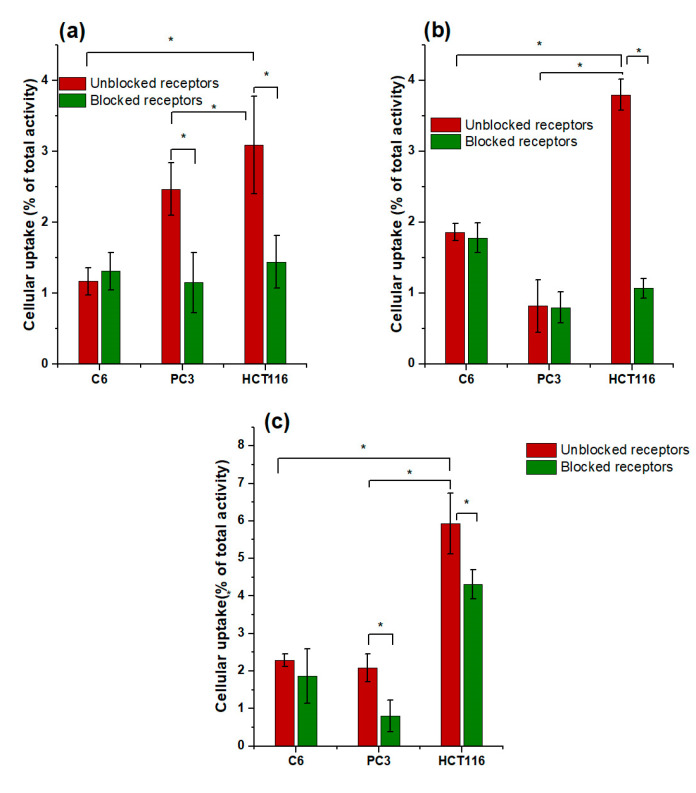
In vitro (**a**) ^225^Ac-iPSMA, (**b**) ^225^Ac-RGD and (**c**) ^225^Ac-iPSMA-RGD uptake in C6 glioma cancer cells (α_V_β_3_ expression), PC3 prostate cancer cells (PSMA and α_V_β_3_ expression), and HCT116 colorectal cancer cells (PSMA and α_V_β_3_ expression) (red bar) (n = 6). In vitro (**a**) ^225^Ac-iPSMA, (**b**)^225^Ac-RGD and ^225^Ac-iPSMA-RGD uptake in C6, PC3, and HCT116 cells with blocked receptors using an excess of the unlabeled peptide (1 mM iPSMA, 1mM RGD, 1 mM iPSMA-RGD) (green bar) (n = 6). * Significant difference, *p* < 0.05; Student’s *t*-test.

**Figure 5:**
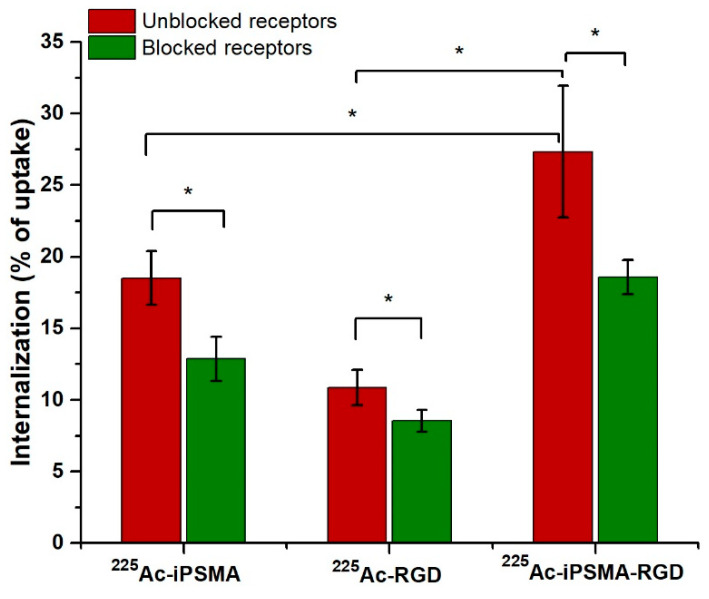
Specific internalization of ^225^Ac-iPSMA, ^225^Ac-RGD, and ^225^Ac-iPSMA-RGD in HCT116 cells. Cells with blocked receptors were co-incubated with an excess of the unlabeled peptide (1 mM iPSMA, 1 mM RGD, 1 mM iPSMA-RGD) (n = 6). * Significant difference, *p* < 0.05; Student’s *t*-test.

HCT116 colorectal cancer cells are PSMA positive (308 nTPM: normalized transcript per million) with low α_V_β_3_ expression (0.2 nTPM). PC3 cells also express PSMA (267 nTPM) and α_V_β_3_ integrin (1.2 nTPM) [6]. Mouse C6 glioma cancer cells are α_V_β_3_ integrin positive with PSMA expression [23,24]. Therefore, the results of ^225^Ac-iPSMA-RGD cellular uptake correlated with α_V_β_3_ and PSMA expression in the three cell lines (Figure 4). Indeed, significantly higher radiopharmaceutical uptake was observed in colorectal cancer cells compared to C6 and PC3 cells, mainly due to the highest PSMA expression (Figure 4), which is the reason why the HCT116 cell line was selected for subsequent in vitro and in vivo studies. Furthermore, the specificity of ^225^Ac-iPSMA-RGD was confirmed by a blocked receptor assay using the monospecific compounds ^225^Ac-iPSMA and ^225^Ac-RGD for comparison with the bispecific radiopharmaceutical ^225^Ac-iPSMA-RGD. The effect of blocked receptors on HCT116 cells (incubation with unlabeled 1-mM iPSMA, RGD and iPSMA-RGD) reduced the density of receptors available for cellular uptake and internalization of ^225^Ac-iPSMA-RGD (0.4 mM). This partially blocked uptake resulted in a statistically significant difference between blocked and unblocked cell receptors due to specific receptor-mediated binding (Figure 5).

**Figure 6:**
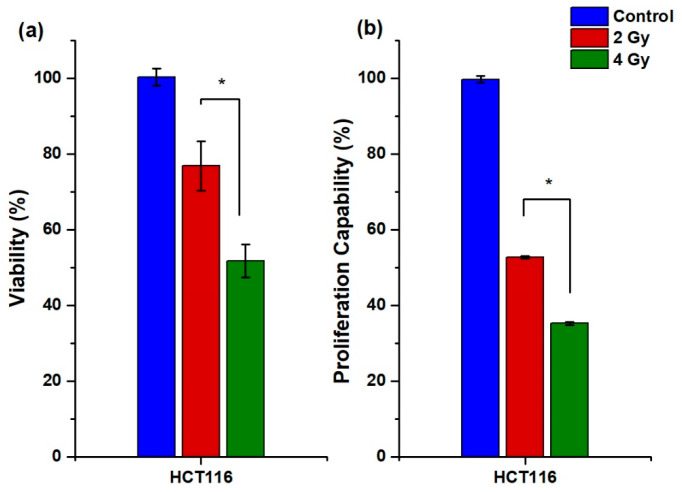
(**a**) Viability at 24 h (n = 6) and (**b**) proliferative capacity (n = 3) of HCT116 cell line after treatment with 2 Gy and 4 Gy of ^225^Ac-iPSMA-RGD, * Significant difference, *p* < 0.05.

The viability assay of ^225^Ac-iPSMA-RGD in the HTC116 cell line indicated a dose-dependent decrease in cell viability from 2 to 4 Gy after radiopharmaceutical exposure, with HCT116 viability being most affected at 4 Gy due to the high cell uptake and internalization of ^225^Ac-iPSMA-RGD, as shown in Figure 6a.

#### 2.3.3. Proliferation Capability Assay

The effect of ^225^Ac-iPSMA-RGD treatment on the ability of HCT116 cells to form colonies (proliferation capability) in clonogenic assays was evaluated in triplicate. Both 2 Gy and 4 Gy radiation doses reduced the proliferation capability in HCT116 cells treated with ^225^Ac-iPSMA-RGD (Figure 6b). Treatment with increasing doses of ^225^Ac-iPSMA-RGD reduced the proliferation capability of HCT116 cancer cells (Figure 6b) in a dose-dependent manner, consistent with PSMA overexpression [25]. Indeed, alpha therapy strongly reduced the proliferation capability (65%) in HCT116 cells (Figure 6b). Similar results were obtained with two ligands, ^225^Ac-mcp-M-alb-PSMA, and ^225^Ac-mcp-D-alb-PSMA, evaluated in the LNCaP cell line. Both ligands reduce colony formation by approximately 50% in a dose-dependent manner, from 1.5 kBq/mL to 0.5 kBq/mL [26].

#### 2.3.4. Apoptosis

**Figure 7:**
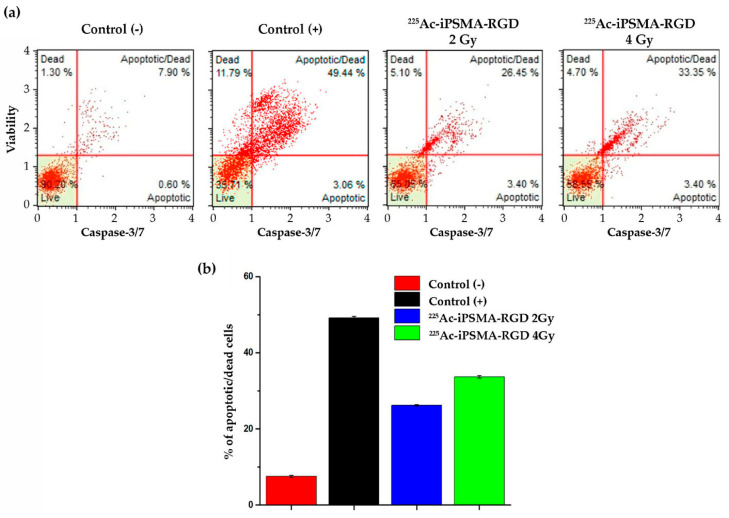
(**a**) Caspase 3/7 and 7-AAD analysis of HCT116 cells treated with 2 Gy and 4 Gy of ^225^Ac-iPSMA-RGD. (**b**) Radiopharmaceutical treatment significantly increased the percentage of HCT116 cells in late apoptosis. Comparison of HCT116 cancer cells percentage in late apoptosis (apoptotic/dead) Caspase 3/7 (+) 7-AAD (+) after ^225^Ac-iPSMA-RGD treatment, cells without any treatment as a negative control, and cells after 24 h nutrient starvation as a apoptosis-positive control (n = 3, *p* < 0.05).

Alpha radiation therapy damages cancer cells in the immediate vicinity by direct or indirect irreversible ionization of DNA, which induces apoptosis [24]. The type of cell death was evaluated to confirm cancer cells’ decreased viability and proliferative capacity after treatment with ^225^Ac-iPSMA-RGD. Cancer cells were treated, and the apoptosis Muse^TM^ Caspase-3/7 kit was used. The HCT116 cell line showed a significant increase in apoptotic/dead cells of approximately 24.6% at a dose of 2 Gy and a 31.2% at a dose of 4 Gy (Figure 7a). Similar results were obtained with ^225^Ac-labeled substance P, the ligand of the neurokinin-1 receptor, which is overexpressed in glioma cells. After 24 h of treatment, the radiopharmaceutical increased apoptotic cells by 25% only in cells overexpressing the substance P receptor, whereas cells not expressing the neurokinin-1 receptor were not affected [27].

#### 2.3.5. Oxidative Stress

**Figure 8:**
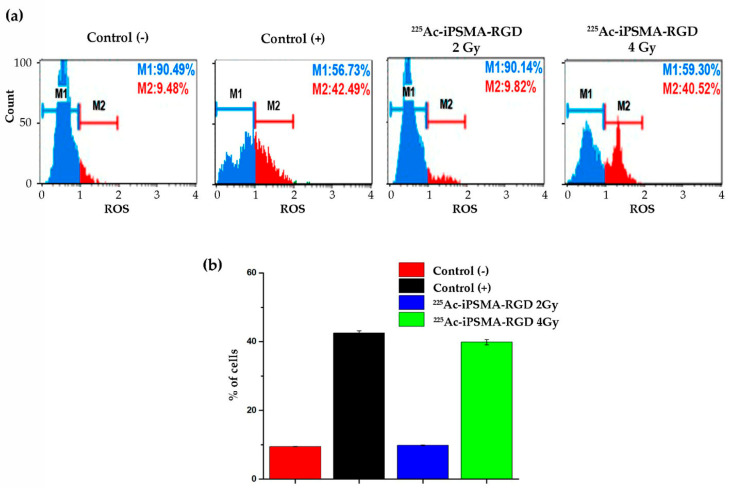
(**a**) Representative plots of the percentage of reactive oxygen species (ROS) produced on HCT116 cancer cells after ^225^Ac-iPSMA-RGD treatment with doses of 2 Gy and 4 Gy. (**b**) Comparison of HCT116 cancer cells percentage in oxidative stress (based on the intracellular detection of superoxide radicals) ROS (+) after ^225^Ac-iPSMA-RGD treatment, cells without any treatment as a negative control, and cells treated with H_2_O_2_ (40 nM) for 30 min as a oxidative stress-positive control (n = 3, *p* < 0.05).

To investigate the mechanism by which ^225^Ac-iPSMA-RGD decreases the viability and proliferative capacity of cancer cells, the generation of oxidative stress after treatment was evaluated. Tumor cells produce elevated levels of reactive oxygen species compared to normal cells. Cancer cells adapt their metabolism to increase intracellular levels of reactive oxygen species to maintain survival and proliferation during tumorigenesis. However, higher levels of reactive oxygen species promote genomic instability, which induces the activation of cell death and significantly decreases resistance to treatment [28]. Indeed, after treatment with a radiation dose of 4 Gy, an increase of approximately 30% of PSMA-positive HCT116 cells with oxidative stress was promoted (Figure 8a). Alpha particles have a tissue range of less than 0.1 mm. This allows the selective elimination of tumor cells by inducing DNA damage through water ionization, which generates reactive oxygen species. This causes several types of damage that are more difficult to repair, especially double-stranded DNA breaks, which explains the therapeutic efficacy of alpha particles [29].

#### 2.3.6. Double-Stranded DNA Breaks

**Figure 9:**
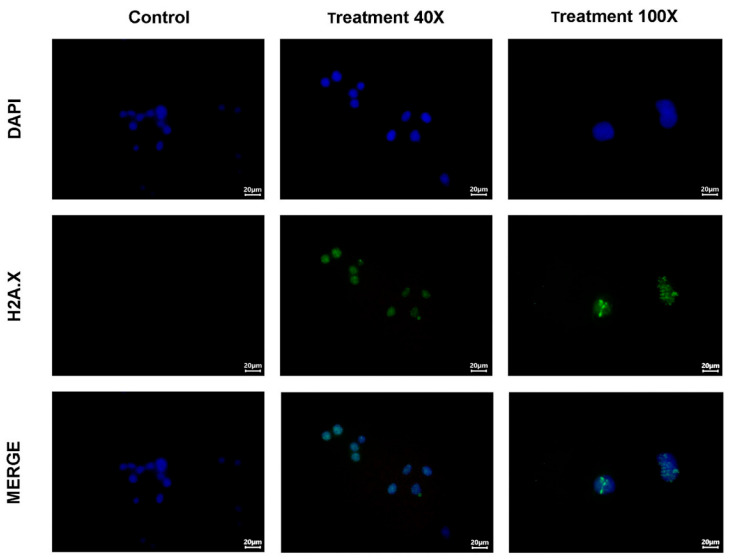
Representative fluorescence microscope images showing phosphorylated H2A.X in the nucleus stained with DAPI of HCT116 cancer cells treated with ^225^Ac-iPSMA-RGD.

^225^Ac-iPSMA-RGD generates alpha particles with high linear energy transfer that can cause lethal double-stranded DNA breaks. Upon induction of DNA damage, the nucleosomal histone protein H2A.X is rapidly phosphorylated at serine 139 at the break site, making phosphorylated H2A.X a sensitive marker for detecting double-stranded DNA breaks [30]. Treatment of HCT116 cells with ^225^Ac-iPSMA-RGD induced high levels of histone H2A.X phosphorylation (Figure 9). Similar results were reported with ^225^Ac-DOTATOC and ^177^Lu-DOTATOC at concentrations of 2.5 to 10 kBq/mL and 0.6 to 10 MBq/mL after 24 and 48 h, respectively. The number of phosphorylated H2A.X foci showed similar levels of double-stranded DNA breaks; however, the mean number of phosphorylated H2A.X foci was 25 to 10 kBq/mL of ^225^Ac-DOTA-TOC and 22 to 10 MBq/mL of ^177^Lu-DOTATOC 48 h after incubation [31]. In this study, the 2 Gy and 4 Gy of ^225^Ac-iPSMA-RGD showed an average of 19 foci of phosphorylated H2A.X in the HCT116 cell line (Figure 9).

### 2.4. In Vivo Assessment

#### 2.4.1. Biodistribution and Biokinetics

**Figure 10:**
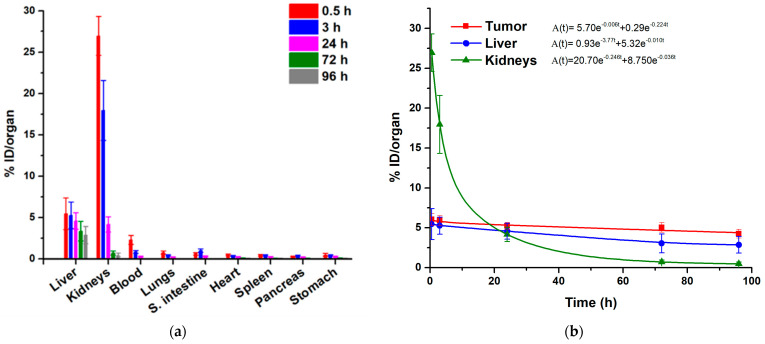
(**a**) Biodistribution pattern in healthy mice of ^225^Ac-iPSMA-RGD; (**b**) Biokinetic models of ^225^Ac-iPSMA-RGD in source organs (liver and kidney) and the malignant tissue of HCT116 tumor-bearing mice.

The ^225^Ac-iPSMA-RGD biodistribution pattern in healthy mice indicated rapid blood clearance with hepatobiliary and renal elimination and no accumulation in other tissue (Figure 10).

The ^225^Ac-iPSMA-RGD biokinetic model showed that most of the activity captured in the liver is cleared slowly (λ = 0.01), with a half-life of 69 h (t_1/2_ = ln2/λ = 69 h), possibly related to the fraction retained in the reticuloendothelial system and to the presence of Ac^3+^ and its progeny released from the peptide radiocomplex, while a small amount of activity is rapidly cleared (λ = 3.77; t_1/2_ = 0.18 h) via hepatobiliary elimination. The relatively short half-life observed in the renal biokinetic model (t_1/2_ = ln2/0.246 = 2.81 h) was correlated with renal elimination and proximal tubule reabsorption, whereas the slow elimination (t_1/2_ = ln2/0.036 = 19.3 h) could be related to some in vivo degradation of the peptide molecule in the kidney, with prolonged retention of the ^225^Ac-peptide fragment [32]. The largest number of nuclear transitions (N) and the longest retention time of ^225^Ac-iPSMA-RGD was observed in the tumor (t_1/2_ = ln2/0.006 = 116 h).

**Figure 11:**
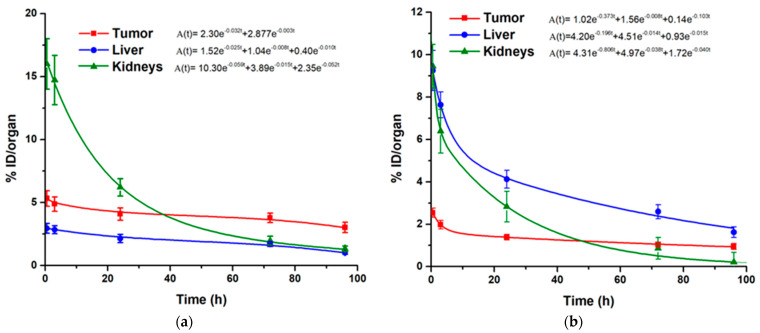
Biokinetic models of (**a**) ^225^Ac-iPSMA and (**b**) ^225^Ac-RGD in source organs (liver and kidney) and the malignant tissue of HCT116 tumor-bearing mice.

For comparative purposes, the biokinetic models of the monomers ^225^Ac-RGD and ^225^Ac-PSMA were also obtained (Figure 11), which showed lower accumulation and nuclear transitions in the tumor compared to ^225^Ac-iPSMA-RGD and, consequently, lower tumor radiation absorbed doses in mice treated with the monomeric ^225^Ac-radiopharmaceuticals (Table 1). These results were expected due to the molecular recognition advantages of dual targeting radiopeptides, as previously reported [23,33].

It is also important to note that the absorbed radiation dose delivered to the tumor mass of mice treated with ^225^Ac-iPSMA-RGD was thirteen times that given to the kidney, and fifteen times that to the liver (Table 1). In contrast, the tumor-to-kidney and tumor-to-liver radiation dose ratios were lower for ^225^Ac-RGD and ^225^Ac-PSMA compared to ^225^Ac-iPSMA-RGD (Table 1).

**Figure 12:**
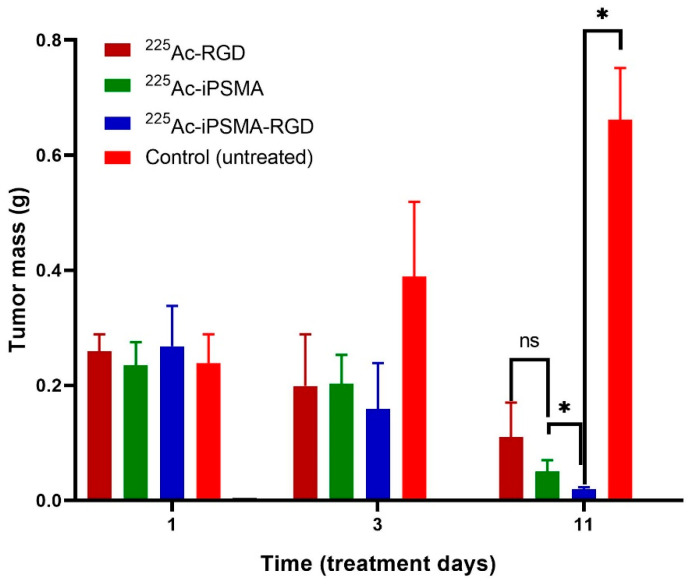
Tumor size progression in HCT116 tumor-bearing mice on different days after treatment with 37 kBq of ^225^Ac-iPSMA, ^225^Ac-RGD, or ^225^Ac-iPSMA-RGD. The group of untreated mice was considered as a control. * Statistically significant difference (*p* < 0.05), ns: no statistically significant difference (*p* < 0.05).

The tumor size progression in mice treated with 37 kBq of ^225^Ac-iPSMA, ^225^Ac-RGD, or ^225^Ac-iPSMA-RGD was significantly lower (*p* < 0.05) compared to that of the control group (untreated mice) (Figure 12). Eleven days after the administration of the treatments (after one ^225^Ac half-life), tumor size in the ^225^Ac-iPSMA-RGD group was also significantly lower (*p* < 0.05) compared to ^225^Ac-RGD and ^225^Ac-PSMA groups (Figure 12). These tumor size data correlate with the ablative radiation doses of the ^225^Ac radiopharmaceuticals in the malignant tissues (Table 1 and Figure 12). Importantly, the application of 7400 kBq of ^225^Ac-PSMA-617 in humans is sufficient to achieve ablation of extensive tumor masses in patients with metastatic prostate cancer [34]. Thus, the results obtained with 37 kBq of ^225^Ac in mice bearing HCT116 colon cancer tumors indicate the ablative potential of ^225^Ac-iPSMA-RGD in the treatment of colorectal cancer.

Although biodistribution studies were used to assess dosimetry (%ID in whole organs was calculated), ^225^Ac in bone was also measured, but it was low (0.5 ± 0.3%ID/g).

**Figure 13:**
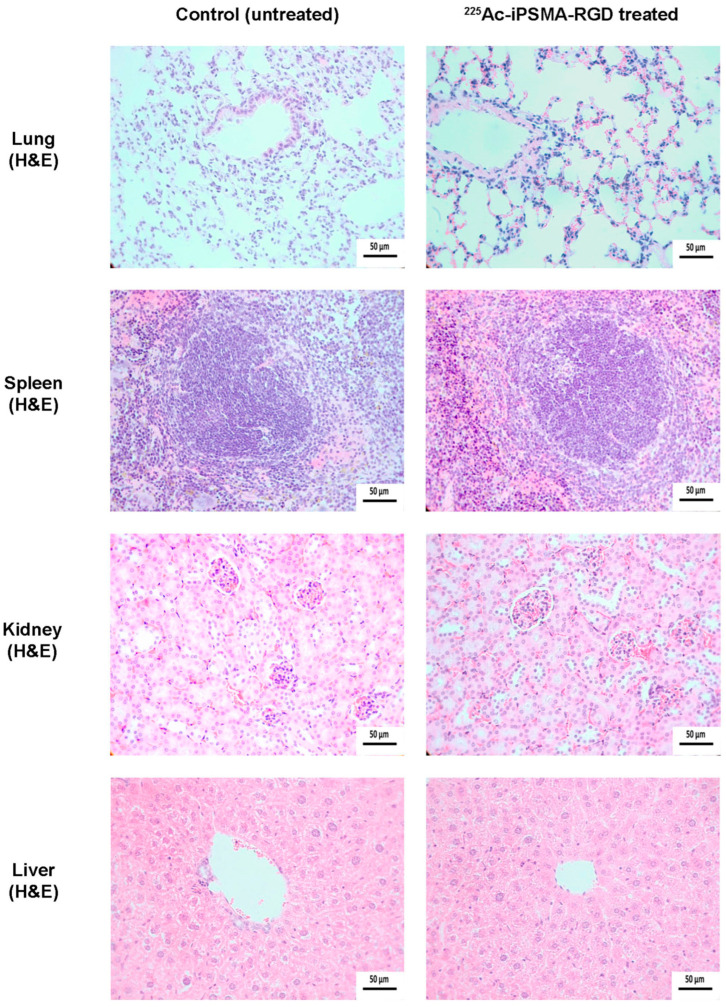
Microscopic views (magnification 400×) of lung, spleen, kidney, and liver tissues from mice exposed to ^225^Ac-iPSMA-RGD and from untreated mice (control group). Histological staining (H&E) evaluation showed no pathologic changes in the organs of treated and untreated mice. No evidence of cytotoxic degeneration was observed in cell tissues.

**Figure 14:**
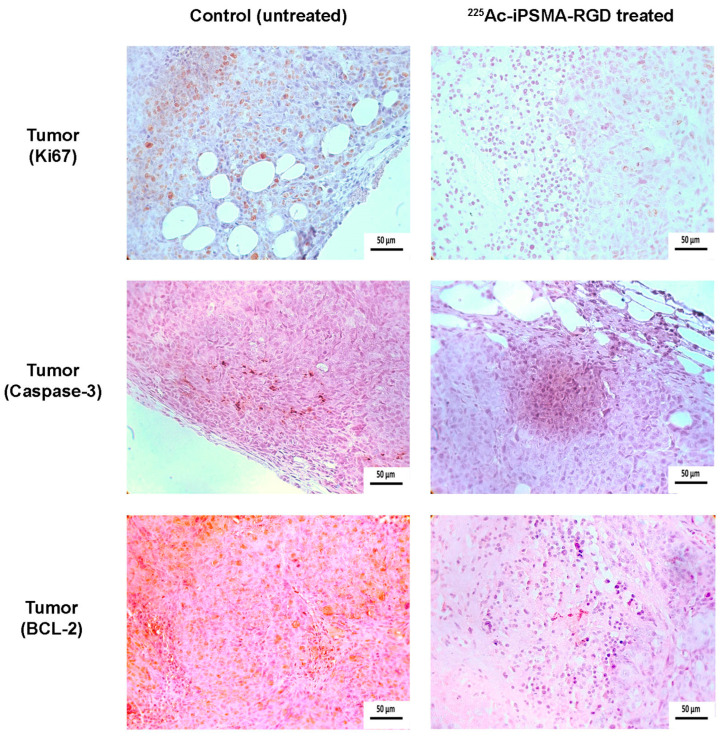
Microscopic views (magnification 40×) of tumor from mice treated with ^225^Ac-iPSMA-RGD and from untreated mice (control group). Histological staining (H&E) evaluation of tumor cell proliferation activity (Ki67 expression; brown staining) was significantly lower in mice treated with ^225^Ac-iPSMA-RGD than that of tumors in untreated mice. ^225^Ac-iPSMA-RGD treatment inhibited Bcl-2 expression (brown staining) and increases Caspase-3 cleavage (brown staining).

The serum levels of creatinine (0.215 ± 0.071 mg/dL), alanine aminotransferase (70 ± 8 IU/L), and aspartate aminotransferase (141 ± 19 IU/L) in mice on day 11 after treatment with ^225^Ac-iPSMA-RGD were not significantly different (*p* < 0. 05) from those obtained in control (untreated) mice: 0.197 ± 0.065 mg/dL, 75 ± 6 IU/L, and 144 ± 23 IU/L for creatinine, alanine aminotransferase, and aspartate aminotransferase, respectively. These results indicated that no damage to renal or hepatic function was detected, which was confirmed by histopathologic studies (Figure 13). In contrast, the tumor proliferative activity (Ki67 expression) of ^225^Ac-iPSMA-RGD-treated mice was significantly lower than that of tumors in untreated mice (control group). Additionally, treatment with ^225^Ac-iPSMA-RGD inhibited the Bcl-2 expression and upregulated the Caspase-3 activity, which is consistent with the apoptosis increasing in the treated tumor (Figure 14).

#### 2.4.2. Ex-Vivo Imaging

**Figure 15:**
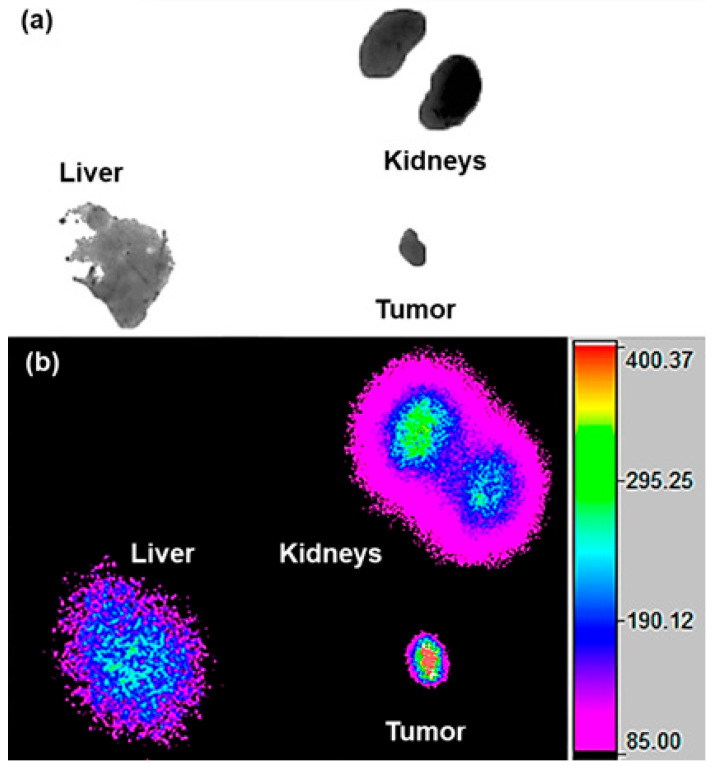
Ex vivo (**a**) transmission/(**b**) radioisotopic images of an HCT116 tumor-bearing mouse eleven days after ^225^Ac-iPSMA-RGD administration (37 kBq).

Ex-vivo radioisotopic imaging of HCT116 tumor-bearing mice 11 days after ^225^Ac-iPSMA-RGD administration (37 kBq) confirmed that the tissue accumulating the radiotracer for the longest time is the tumor, while the kidneys and liver eliminate most of the radiotracer, according to their physiological function (Figure 15).

## 3. Materials and Methods

The heterodimeric HYNIC-iPSMA-RGD (OH-Glu-CO-Lys(2Nal-Cys[ciclo(Arg-Gly-Asp-dPhe-Lys(GMBS)-HYNIC-OH) (GMBS: N-(gamma-maleimidebutyryloxy MW 1524.09 g/mol) peptide was designed at LANIDER, ININ (Instituto Nacional de Investigaciones Nucleares, Estado de México, Mexico) and the synthesis was required to Ontores Biotechnologies Co., Ltd. (Hangzhou, China). Dimethylformamide (DMF), diisopropylethylamine (DIPEA), p-SCN-Benzene-HEHA (2,2′,2″,2‴,2′‴,2″‴-(2-(4-isothiocyanate benzyl)-1,4,7,10,13,16-hexaazacyclooctadecane-1,4,7,10,13,16-hexayl)hexaacetic acid), MW 753.30 g/mol, were obtained from Macrocyclics (Dallas, TX, USA). Hydrochloric acid (99.999% trace metal basis) was purchased from Merck (KGaA, Darmstadt, Germany). The 2,3-bis-(2-methoxy-4-nitro-5-sulfophenyl)-2H-tetrazolium-5-carboxanilide) (XTT) kit was obtained from Roche Diagnostics (Indianapolis, IN, USA) and the HCT116, C6 and PC3 cell lines were obtained from ATCC (Atlanta, GA, USA). RPMI media and Fetal Bovine Serum (FBS) were purchased from Bovine and Biology (Mexico City, Mexico).

^225^Ac was purchased as ^225^Ac(NO_3_)_3_ form from van Overeem Nuclear bv (Amsterdam, The Netherlands). The purchase was partially funded by the IAEA.

### 3.1. Conjugation of HYNIC-iPSMA-RGD to HEHA-SCN

For conjugation of p-SCN-Benzene-HEHA (HEHA) to HYNIC-iPSMA-RGD, 3.0 mg (1.3 µmol) of HYNIC-iPSMA-RGD was dissolved in 200 µL of 0.2 M sodium bicarbonate buffer, pH 9.5. The HEHA (0.9 mg, 1.2 µmol) was also dissolved in 200 µL of bicarbonate buffer 0.2 M pH 9.5. Both solutions were mixed and stirred for 24 h at 37 °C.

The HEHA-HYNIC-iPSMA-RGD was purified using Tube-O-Dialyzer (Medi, 1 KDa MWCO, Biosciences, St Louis, MO, USA), followed by an additional HPLC purification. A flow rate of 1 mL/min of a gradient system (solvent A: Acetonitrile-TFA 0.1% and Solvent B: Water-TFA 0.1%) was used according to the following scheme: from minute 1 to 3, 100% of B was maintained, from min 3 to 10, 50% of B was reached and from 10–20 min 50% was maintained. From 20–23 min, the system reached 70% of B. At min 27, 100% of B was continued until 30 min. Purified compounds were lyophilized for further characterization and radiolabeling.

For comparative purposes, HEHA was also conjugated to the monomer HYNIC-iPSMA and HYNIC-RGD. Briefly, to prepare HEHA-HYNIC-iPSMA (HYNIC-iPSMA), 2.0 mg (3 µmol) of HYNIC-iPSMA was reacted with 2.8 µmol (2.1 mg) of HEHA. For the synthesis of HEHA-HYNIC-RGD (HEHA-RGD), 3.0 mg (2 µmol) of HYNIC-RGD was dissolved in 200 µL of 0.2 M sodium bicarbonate buffer, pH 9.5. The HEHA (0.9 mg, 1.2 µmol) was dissolved in 200 µL of sodium bicarbonate buffer 0.2 M pH 9.5. Both solutions were mixed and stirred for 24 h at 37 °C.

### 3.2. Spectroscopic Characterization by FT-IR and UV-Vis Characterization

The HEHA, iPSMA-RGD, and HEHA-iPSMA-RGD were analyzed in solid form by attenuated total reflection FT-IR spectroscopy in lyophilized form using an Agilent Technologies FT-IR 660 spectrometer (50 scans collected at a resolution of 0.4 cm^−1^).

For UV-Vis characterization, the starting materials and the final conjugate spectra were acquired in a UV/Vis spectrophotometer Genesys 10S (Thermofisher Scientific, Waltham, MA, USA), from 210 to 400 nm.

### 3.3. Mass Spectrometry Characterization

Analysis was performed on an ADQUITY UPLC H-Class with a QDa mass detector (Waters Corporation, Milford, CT, USA).

### 3.4. Radiolabeling and Radiochemical Purity Evaluation

^225^Ac(NO_3_)_3_ 14.5 MBq was dissolved by adding 900 μL of 0.08 M HCl to the original vial. The vial was sterilized by autoclaving (121 °C for 15 min). Then, 1100 μL of 1M sodium acetate buffer, pH 5.0, was added, and the stock solution was stored at room temperature for further radiolabeling. Aliquots of 100 μL ($A_{0}=$ 7.25 kBq) were taken and used as needed.

After optimizing the radiolabeling conditions (mass, pH, temperature, reaction time), ^225^Ac-HEHA-HYNIC-iPSMA-RGD (^225^Ac-iPSMA-RGD) was obtained using 60 μg of the final cold conjugate HEHA-HYNIC-iPSMA-RGD (iPSMA-RGD). Briefly, 60 μL of a stock solution (1 mg/mL) was added to a reaction vial, then 50 μL of 1M sodium acetate buffer, pH 5.0, was added and the mixture was heated at 95 °C ± 1 °C for 1 h. After radiolabeling, solid phase extraction (Sep-Pack C-18 cartridge, Waters, USA) was used to purify ^225^Ac-iPSMA-RGD.

To evaluate the radiochemical purity, a reversed-phase HPLC system with Waters Millenium 32 software coupled to an in-line UV-Vis and radioactivity detector was used, as previously detailed. To recover free ^225^Ac and ^225^Ac-HEHA-iPSMA, fractions of 1.0 mL were collected, and the activity quantification was through the 218 keV-gamma emission of Fr-221 by assuming secular equilibrium[9]. The activity was measured in a NaI(Tl) well-type detector (Auto In-v-tron 4010; NML Inc., Houston, TX, USA).

### 3.5. Serum Stability

After adding 200 μL of ^225^Ac-iPSMA-RGD to the diluted human serum (1:5, *v*/*v*) in a vial (n = 3), the mixture was kept at 37 °C in a dry bath. At 0.5 h, 1 h, and 3 h intervals, 200 μL of each vial was removed and 1 mL of methanol: acetonitrile (1:1, *v*/*v*) was added to precipitate the proteins. The total activity was measured in a NaI(Tl) well-type detector by 218 keV gamma emission of Fr-221 and the solution was centrifuged at 3000 rpm for 5 min. The supernatant was then decanted and the pellet measured to determine ^225^Ac binding proteins. The stability of ^225^Ac-iPSMA-RGD saline solution (0.9% NaCl) and human serum was also evaluated by radio-HPLC as mentioned above.

### 3.6. Cell Culture

For in vitro evaluation, PSMA-positive HCT116 (ATCC-CCL-247) human colon cancer cells and the PSMA-negative control PC3 (CLR-1435) prostatic adenocarcinoma cells and C6 glioma cancer cells were cultured in RPMI media supplemented with L-glutamine, 10% fetal bovine serum, and penicillin-streptomycin (100 U/mL/100 μg/mL) at 37 °C in a 5% carbon dioxide atmosphere.

### 3.7. Uptake and Internalization in Cells

The ^225^Ac-iPSMA-RGD uptake was performed on HCT116, C6 and PC3 cell lines, which were selected based on their variable PSMA and RGD expression according to previous research by our group and others. Each cell line was diluted to 1 × 10^6^ cells with 1 mL of PBS in tubes and incubated at 37 °C with 30 μL of ^225^Ac-iPSMA-RGD (20 μL, 70 Bq) for 0.5 h, 1 h and 3 h (n = 6). At each time point, the tubes were centrifuged at 3000 rpm for 5 min and rinsed twice with PBS. The measured activity in a NaI(Tl) well-type detector in the pellet, represents the uptake and internalization. To remove the ^225^Ac-iPSMA-RGD at the cell membrane level, 1 mL of 0.2 M CH_3_COOH /0.5 M NaCl was added and the pellet was resuspended. The tubes were then centrifuged and washed with PBS twice (this activity is considered internalization). Since the HCT116 cell line demonstrated the highest uptake, it was selected for the subsequent in vitro and in vivo evaluation.

To demonstrate specificity, PSMA and αvβ3 integrin receptors were blocked in HCT116 cells with cold iPSMA, RGD, and iPSMA-RGD (1 mM) which were added immediately before the addition of ^225^Ac-iPSMA-RGD.

### 3.8. Cytotoxicity after ^225^Ac-iPSMA-RGD Treatment

Cytotoxic assays were performed in triplicate (n = 6) using HCT116 cells. After harvesting each cell line, 10,000 cells per well were placed in a 96-well plate and incubated at 37 °C to allow adherence. After 24 h, the culture medium was removed, and treatments (2 Gy, 4 Gy) were placed in each well. To deliver 2 and 4 Gy of radiation dose, 6 Bq/well and 12 Bq/well were added and exposed for 15 h. Then, the treatments were removed and replaced with the medium. The cytotoxicity was evaluated at 24 h by using the Cell proliferation assay (Roche Diagnostics GmbH, Mannheim, Germany). Statistical analysis was performed with two-way ANOVA followed by Tukey’s multiple comparisons test using GraphPad Prism 5.0 Software. Data are shown as mean ± SEM. A *p* < 0.05 was considered statistically significant.

### 3.9. Proliferation Capability Assay

HCT116 cell line was used to evaluate the ability of a single cell to form colonies after exposure to ^225^Ac-iPSMA-RGD treatment, in triplicate. We seeded 500 cells in each well (six-well plate) and treated them with 4.5 Bq/well (2 Gy), 9.0 Bq/cell (4 Gy), and control (no treatment) for 1 h, dissolved in 2 mL of RPMI medium. After exposure, the treatments and medium were removed and replaced with fresh medium. The plates were incubated for 15 days. Surviving colonies were fixed for 10 min with 4% paraformaldehyde at room temperature and washed with type 1 water. Afterward, they were stained with 0.1% crystal violet for 30 min, washed with purified water, and dried. For quantification, 10% acetic acid was added for 5 min to extract the crystal violet and the absorbance was measured at 590 nm in a microplate absorbance reader (EpochTM; BioTek Instruments, Winooski, VT, USA). Statistical analysis was performed with two-way ANOVA followed by Tukey’s multiple comparisons test using GraphPad Prism 5.0 Software. Data are shown as mean ± SEM. A *p* < 0.05 was considered statistically significant.

### 3.10. Apoptosis

Apoptosis in the colorectal HCT116 cancer cell line was evaluated in triplicate, using the Muse^TM^ Caspase-3/7 kit, Cat No. MCH100108 (Merck Millipore, Burlington, MA, USA). Briefly, cells were seeded in cell culture flasks at a density of 1 × 10^6^ cells and exposed to 4.5 Bq of (2 Gy) and 9.0 Bq for 1 h Bq (4 Gy) of ^225^Ac-iPSMA-RGD. Untreated cells were used as the control group and the positive control was prepared through nutrient starvation for 24 h [35]. First, cells were trypsinized and suspended in 50 µL of 1X Assay Buffer BA (1 × 10^6^ cells/mL). Then, 5 µL of Caspase-3/7 reagent working solution was added at 37 °C with 5% of CO_2_ for 30 min. After incubation, 150 µL of Caspase 7-AAD working solution was added to each tube. Finally, tubes were incubated for 5 min, protected from light, and fluorescent intensities were examined by flow cytometry using a Muse Cell Analyzer (Merck Millipore, Burlington, MA, USA) and were conducted in triplicate).

Statistical analysis was performed with two-way ANOVA followed by Tukey’s multiple comparisons test using GraphPad Prism 5.0 Software. Data are shown as mean ± SEM. A *p* < 0.05 was considered statistically significant.

### 3.11. Reactive Oxygen Species

Reactive oxygen species (ROS) were detected with the Muse™ Oxidative Stress kit, (Merck Millipore, Burlington, MA, USA) by measuring the produced intracellular superoxide radicals through dihydroethidium fluorescence. H_2_O_2_ (40 nM) was used as the positive control. Briefly, cells were seeded in cell culture flasks at a density of 1 × 10^6^ cells and exposed to 4.5 Bq (2 Gy) and 9 Bq (4 Gy) of ^225^Ac-iPSMA-RGD. Untreated cells were used as the control group. First, cells were trypsinized and suspended in 10 μL of 1X Assay Buffer. Then 190 μL of the oxidative stress reagent working solution was added to each cell suspension. The suspension was then gently vortexed and incubated at 37 °C with 5% CO_2_ for 30 min. Fluorescent intensities were examined by flow cytometry using a Muse Cell Analyzer (Merck Millipore, Burlington, MA, USA) and were performed in triplicate. Statistical analysis was performed with two-way ANOVA followed by Tukey’s multiple comparisons test using GraphPad Prism 5.0 Software. Data are shown as mean ± SEM. A *p* < 0.05 was considered statistically significant.

### 3.12. DNA Double-Stranded Breaks Assay

For the evaluation of DNA double-stranded breaks (DSBs), the phosphorylation of the Ser-139 gamma histone variant H2A.X (γH2A.X) was used as a biomarker. HCT116 cells were grown on a glass slide after exposure of 4.5 Bq (2 Gy) and 9.0 Bq (4 Gy) of ^225^Ac-iPSMA-RGD for 60 min. The anti-phospho-histone H2A.X (ser139) cl 6L16 ZooMAb (Cat. ZRB05636, Merck, Darmstadt, Germany) antibody was used as the primary antibody. The Alexa Fluor 488-conjugated Goat anti-Rabbit IgG (H + L) (Invitrogen Cat. A32731) was used for the visualization of phosphorylated H2A.X. Afterwards, the slides were stained with DAPI, and the foci were observed with a Meiji Techno fluorescence microscope (Mod. MT6200: Saitama, Japan). To quantify the foci, clear and easily distinguishable dots were counted. At least 30 cells were imaged per slide, and the average number of foci per cell was calculated. Statistical analysis was performed with two-way ANOVA followed by Tukey’s multiple comparisons test using GraphPad Prism 5.0 Software. Data are shown as mean ± SEM. A *p* < 0.05 was considered statistically significant.

### 3.13. Biodistribution

All animal procedures followed the Ethical Regulations for the Handling of Laboratory Animals (NOM-062-ZOO-1999) and the requirements of the Institutional Animal Care and Use Committee, according to an approved protocol (CICUAL-08-2018-2021). Male Nu/Nu (UPEAL, CINVESTAV, I.PN, Mexico City, Mexico) mice, 6–8 weeks of age, were housed in an aseptic isolation room. Mice were injected with 3.7 kBq (50 µL) of ^225^Ac-iPSMA-RGD and necropsied at 1, 3, 24, 72, and 96 h (n = 3). The liver, heart, kidney, pancreas, lung, spleen, small intestine, and stomach were measured using the Francium-221 photopeak (218 keV) in a hyperpure germanium gamma spectrometer (Ortec, Atlanta, GA, USA) [9]. Results were recorded as a percentage of injected dose (%ID) per organ. Blood and bone samples were also dissected, and the associated activity was recorded as %ID/g. A second group of mice was inoculated subcutaneously with 1 × 10^6^ HCT116 cells. After the mice developed a tumor (0.220 ± 0.030 g), they were treated with 3.7 kBq (50 µL) of ^225^Ac-iPSMA-RGD (n = 3) via tail vein injection. Animals were necropsied on day 11 post-treatment, as described above. Radioisotopic/X-ray ex vivo images of the source organs and tumor were obtained in a preclinical imaging system (Bruker, XTREME, Billerica, MA, USA).

### 3.14. Absorbed Radiation Dose Estimation

The %ID/organ data were used to calculate the biokinetic models. The $A_{h}(t)$ functions were obtained by decay correction of the biokinetic models, i.e., by adding the radioactive constant $(\lambda_{R}$) to the biological constant $(\lambda_{R}$) as follows (Equation (1)):

(1) $$A\left(t\right)={Be}^{-(\lambda_{R}+\lambda_{B})t}+{Ce}^{-(\lambda_{R}+\lambda_{B})t}+{De}^{-(\lambda_{R}+\lambda_{B})t}$$

The ^225^Ac decay chain was considered for dosimetry. The N (number of nuclear transitions) value in each murine organ was calculated by integrating the biokinetic models (from t = 0 to t = 11 days). DF (dose factor, mGy/MBq) values were obtained from OLINDA 2.0 code (Dose, Gy = NxDF).

### 3.15. Histology and Immunohistochemistry Protocol

Formalin-fixed paraffin-embedded samples (lung, spleen, kidney and tumor), were sectioned at 4 µm sections and stained with H&E. For immunohistochemical reactions, samples were deparaffinized, hydrated, and subjected to an antigen retrieval protocol (slides were immersed in a prewarmed Citrate-EDTA buffer solution (10mM citric acid, 2 mM EDTA, 0.05% Tween 20, pH 7.0) at 100 °C for 30 min. Endogenous peroxidase was then blocked with 0.9% hydrogen peroxide (H_2_O_2_) solution for 5 min. To prevent non-specific binding, the samples were incubated with 5% bovine serum albumin (BSA) for 10 min. After subsequent washes with phosphate-buffered saline (PBS)-Tween (0.05%), samples were incubated overnight at 4 °C with primary monoclonal rabbit anti- Ki-67 [SP6] antibody (Biocare Medi-cal-CRM 325A, Pacheco, CA, USA). After subsequent washing, samples were incubated with secondary HRP donkey anti-rabbit IgG (minimal x-reactivity) antibody (Biolegend-406401 San Diego, CA) for 1 h. For mitochondrial permeabilization processes estimation, the samples were incubated overnight at 4 °C with unconjugated Bcl-2 (C2) sc-7882 antibody (Santa Cruz Biotechnology Inc., Dallas, TX, USA). As a secondary antibody, we used m-IgG1 BP-HRP: sc-525408 (Santa Cruz Biotechnology Inc., Dallas, TX, USA). Samples were incubated for 1 h. For apoptotic cell estimation, unconjugated active (cleaved) Caspase-3 Monoclonal Antibody bsm-33199M (Bioss Inc., Woburn, MA, USA) was used. The samples were incubated overnight at 4 °C, and the secondary antibody was goat Anti-Mouse IgG (Fc specific)–peroxidase antibody A2554 (Millipore-Sigma Aldrich, Merck KGaA, Darmstadt, Germany). Samples were incubated for 1 h. Both primary and secondary antibodies were used at a 1:100 dilution. To visualize the immunoreactions, the samples were washed with distilled water and Tris-buffered saline-Tween (TBST) for 5 min, and the diaminobenzidine (DAB) (Dako Liquid DAB+ Substrate Chromogen System K3468) was applied for 60 s. Excess chromogen was removed by washing with PBS-Tween. Finally, the slides were counterstained with Harris hematoxylin, dehydrated, and coverslipped. As a negative control, immunoreactions were performed using the same method with PBS instead of the primary antibody. Images were captured using a Lumenera INFINITY X-32 MP camera in a Carl Zeiss Axiostar microscope.

## 4. Conclusions

The radiotherapeutic dual-targeting ^225^Ac-iPSMA-RGD, synthesized and evaluated in this research, showed cytotoxic, biokinetic, and dosimetric properties suitable for alpha therapy, delivering ablative radiation doses at the tumor microenvironment level. Considering the phenotype of HCT116 cancer cells, the results of this study warrant further dosimetric and clinical studies to determine the potential of ^225^Ac-iPSMA-RGD in the treatment of colorectal cancer.

## Figures and Tables

**Table 1:** Total nuclear transformations (N) and radiation absorbed doses in target organs of athymic mice produced by ^225^Ac-iPSMA, ^225^Ac-RGD, and ^225^Ac-iPSMA-RGD.

Organ	^225^Ac-iPSMA		^225^Ac-RGD		^225^Ac-iPSMA-RGD	
	$N=\int_{t=0}^{t=11d}A\left(t\right)dt\;MBq\cdot h$	Radiation Absorbed Dose Gy/37 kBq	$N=\int_{t=0}^{t=11d}A\left(t\right)dt\;MBq\cdot h$	Radiation Absorbed Dose Gy/37 kBq	$N=\int_{t=0}^{t=11d}A\left(t\right)dt\;MBq\cdot h$	Radiation Absorbed Dose Gy/37 kBq
Kidney	4.41	22.04	1.78	8.90	3.52	17.63
Liver	2.10	6.38	3.92	11.89	5.23	15.85
Tumor	6.04	182.87	1.72	51.92	7.83	237.07

## Data Availability

Data are contained within the article.
